# Tongaline and Yellow Fever

**Published:** 1905-10

**Authors:** 

**Affiliations:** K. D. Mellier, Secy., St. Louis, Mo.


### Tongaline and Yellow Fever.

**The Mellier Drug Company of St. Louis Explains and Sets Itself Right.**

*Editor Buffalo Medical Journal:*

SIR:—We hand you herewith a copy of a reading notice which recently appeared in a number of medical journals. We regret that its meaning has been misconstrued so that some physicians have imagined that we had recommended tongaline for yellow fever.

On page 936 of the September 23rd *Journal of the American Medical Association* this fact has been published under the heading of "Tongaline and Yellow Fever." We would state most emphatically that nothing was farther from our minds than the idea of suggesting the use of tongaline in yellow fever and this is evident from the fact that the word "parasites" in the notice must refer to the malarial germs and not to the stegomyia fasciata.

We would ask you therefore to please print the enclosed copy headed "Important Notice" in the very next issue of your journal, as you will readily observe that we wish to correct an erroneous impression as early as possible.

Thanking you in advance for your kindness in complying with this request, we remain,

Very respectfully yours,

MELLIER DRUG COMPANY,

St. Louis, Mo., September 27, 1905. K. D. Mellier, Secy.

---

IMPORTANT NOTICE.

Judging from communications recently received, our reference to the "stegomyia fasciata" in connection with the "stegomyia punctata" has caused some physicians to suppose that we recommended tongaline for yellow fever. This we emphatically disclaim. The mention of these two species of mosquitoes was for the purpose of indicating that the mode of inoculation of yellow fever and malaria was precisely the same, and a careful reading of our statement will show that we had no intention to suggest that tongaline was indicated in yellow fever, but on account of its pronounced eliminative action it did possess decided therapeutic value in the treatment of malaria. We regret exceedingly that the notice referred to should have been misunderstood or misconstrued by anybody.

MELLIER DRUG COMPANY,

St. Louis

